# RNA‐Sequencing Reveals Two Subgroups of Eccrine Porocarcinomas and Poromas

**DOI:** 10.1111/jcmm.71140

**Published:** 2026-04-14

**Authors:** Maya Puttonen, Sami Kilpinen, Maria von Willebrand, Kalle Ojala, Tom Böhling, Virve Koljonen, Harri Sihto

**Affiliations:** ^1^ Department of Pathology University of Helsinki and Helsinki University Hospital Helsinki Finland; ^2^ Molecular and Integrative Biosciences Research Programme University of Helsinki Helsinki Finland; ^3^ HUS Vatsakeskus Helsinki University Hospital Helsinki Finland; ^4^ Department of Plastic Surgery University of Helsinki and Helsinki University Hospital Helsinki Finland

**Keywords:** eccrine porocarcinoma, eccrine poroma, oncology, RNA sequencing, skin cancer

## Abstract

Eccrine porocarcinoma (EPC), a rare malignant eccrine gland tumour, remains molecularly understudied. Transcriptomic studies of EPC and benign eccrine poroma (EP) have identified recurrent fusions and expression changes, but differences distinguishing malignant EPC from benign EP are unclear. RNA was extracted from formalin‐fixed, paraffin‐embedded (FFPE) samples (13 EPCs and 49 EPs) from Helsinki Biobank and Finnish Clinical Biobank Tampere. RNA sequencing characterized transcriptomic profiles. Histopathological features were assessed on haematoxylin–eosin–stained sections, and fusion genes were evaluated by NUT and YAP1 immunohistochemistry. EPCs and EPs clustered into two transcriptomic groups regardless of tumour subtype, primarily distinguished by differential expression of genes involved in skin metabolism. The metabolism‐high group showed higher expression of genes associated with immune‐related processes, mesothelin, and Ras‐MAPK signalling. The metabolism‐low group contained a subgroup enriched for Hedgehog pathway–associated genes, such as *GLI1*, *GLI2*, *HHIP*, *LRP2*, and *PTCH2*. All samples with the *YAP1–NUTM1* fusion pattern belonged to the metabolism‐low group and showed elevated *NUTM1* expression in the heatmap. RNA sequencing revealed transcriptomic subgroups in EPC/EP partly linked to fusions. The results underscore the necessity for further investigation into the disrupted signalling pathways, which may facilitate the development of targeted therapies.

**Abbreviations**
DPA: Digital papillary adenocarcinoma
EPC: Eccrine porocarcinoma
EP: Eccrine poroma
FDA: U.S. Food and Drug Administration
FFPE: Formalin‐fixed, paraffin‐embedded
GLI: Glioma‐associated oncogene
HH: Hedgehog
HPV: Human papillomavirus
IHC: Immunohistochemistry
PTCH: Patched
QC: Quality control
SMO: Smoothened
WES: Whole‐exome sequencing

## 1 Introduction

Eccrine porocarcinoma (EPC) is a rare malignant adnexal tumour of the skin that originates from the eccrine sweat gland. Some EPCs develop from its benign counterpart, eccrine poroma (EP) [1]. The incidence of EPC ranges from 0.04 to 0.25 per 100,000 person‐years, depending on the study [2, 3, 4]. EPC and EP are usually treated surgically, and the prognosis of local disease is generally good [5]. However, patients with advanced EPC have limited systemic therapy options [6] and have a high mortality rate of 60%–70% for metastasized disease [5]. Knowledge on the transcriptomic landscape of EPC and EP has recently increased due to accumulating research findings. The first combined whole‐exome sequencing (WES) and RNA sequencing study on an EPC sample reported a missense mutation and decreased expression of *TP53* and altered p53 protein levels [7]. In 2019, the first RNA sequencing project involving EP samples was published, unveiling novel fusion genes such as *YAP1*–*MAML2* (found in 88.5% of the EP samples), *MAML2*–*YAP1* (52.9%), *YAP1*–*NUTM1* (20.2%), and *WWTR1–NUTM1* (1.0%) [8]. Additionally, *YAP1* fusions were also detected in 63.6% of EPC samples [8]. These findings were followed by multiple reports confirming *YAP1*–*NUTM1* gene fusions in EPCs [9, 10], suggesting that fusion genes such as *YAP1*–*NUTM1* may play pivotal roles as driver mutations in both EPs and EPCs. Additionally, recurrent fusions involving the *PAK2* gene were identified in EPs [11].

As with many skin cancers, UV radiation is considered one of the contributing factors in the tumorigenesis of EPCs and EPs. Additionally, emerging evidence suggests a potential link between certain viruses and the development of skin cancer. Such associations include Merkel cell polyomavirus with Merkel cell carcinoma [12], human papillomavirus (HPV) with cutaneous squamous cell carcinoma in immunocompromised individuals [13], and HPV42 with digital papillary adenocarcinoma [14]. Efforts have also been made to investigate the potential involvement of viruses in the aetiology of EPCs and EPs. Previous studies have detected a low copy number of viral DNA of Merkel cell polyomavirus in EPC, suggesting that Merkel cell polyomavirus is merely a passenger virus in EPCs and EPs [15, 16]. Similarly, an association between HPV DNA and EPC has been studied, but no convincing evidence has been found to support HPV involvement in the development or progression of EPCs [17].

Here, the transcriptomic profiles of EPCs and EPs were characterized using RNA sequencing, with viral mRNA load assessed within these profiles. *YAP1* and *NUTM1* fusions were assessed by immunohistochemistry (IHC), alongside IHC for the HPV E7 oncoprotein, which detects the most common high‐risk HPC strains 16, 18, and 45, to characterize HPV's potential role in these tumours. While fusion‐based subgroups have been described previously, to our knowledge, this is the first study to identify distinct transcriptomic subgroups of EPCs and EPs.

## 2 Materials and Methods

### 2.1 Patient Samples

Archival tissue specimens preserved as formalin‐fixed, paraffin‐embedded (FFPE) blocks were obtained from Helsinki Biobank for 13 EPCs and 49 EPs diagnosed between 1987 and 2013 as described in our previous study [18]. Corresponding clinical information was also retrieved from the biobank repository. All EPC specimens, except for two recurrences, were primary tumours. A review of the diagnosis by an experienced pathologist (Tom Böhling) resulted in the reclassification of one specimen, initially diagnosed as an EPC, as a digital papillary adenocarcinoma (DPA). Therefore, there was only one recurrent EPC as this DPA sample was a recurrence from a former excision. The DPA sample was retained in the analysis primarily as a positive control for viral integration analysis, as DPAs are known to contain the HPV42 virus. Additionally, two EPC FFPE samples were collected from Finnish Clinical Biobank Tampere [approval no. BB2018‐006]. The FFPE samples were subjected to RNA extraction. Five FFPE blocks from healthy skin samples were also obtained and subjected to RNA extraction. The institutional ethics committee of Helsinki University Central Hospital has approved this study [HUS/358/2018]. Although the FFPE samples were collected from the same Finnish cohort, neither the DNA material obtained and analysed in our previous study nor the data generated therein were used in the present study.

### 2.2 RNA Extraction From FFPE Samples

Two to three 1‐mm tumour cores were collected from FFPE blocks using an automated tissue microarrayer (TMA Grand Master, 3DHISTECH Ltd., Budapest, Hungary) for subsequent RNA extraction. RNA extraction from FFPE samples was performed with a QIAsymphony SP instrument and QIAsymphony RNA extraction kit (catalogue number 931636, QIAGEN GmbH, Hilden, Germany) according to the manufacturer's instructions. The quality and quantity of the extracted RNA were measured using a 2100 Bioanalyzer (Agilent technologies, California, USA).

### 2.3 Sample Selection for RNA Sequencing

RNA was successfully obtained from 14 EPCs, 36 EPs, 1 DPA, and 5 normal skin samples. The mean RNA concentration was 28.0 ng/μl (range 2.3–186.8 ng/μl). From the 36 EP RNA samples, 20 samples with the highest concentrations were chosen for further analysis, along with all EPC and normal skin samples. The mean RNA Integrity Number value of the samples selected for RNA sequencing was 2.8 (range 1–8.5). However, RNA samples from 5 EPCs, 4 EPs, and 2 normal skin samples failed the quality control (QC) test and were subsequently excluded from RNA sequencing; thus 9 EPCs, 16 EPs, 1 DPA, and 3 normal skin samples remained in the series.

### 2.4 3′ RNA Sequencing and Processing of the 3′ RNA Sequences

RNA extracted from 9 EPC, 16 EP, 1 DPA, and 3 normal skin FFPE samples was 3′ sequenced in the sequencing unit of the Institute of Molecular Medicine Finland as described previously [19]. QC analysis was performed using LabChip GX (Revvity, Waltham, MA, USA). RNA sequencing library was prepared using a QuantSeq 3′ mRNA‐Seq kit (catalogue number 015.96, Lexogen, Vienna, Austria) according to the manufacturer's instructions. A HiSeq 2500 instrument from Illumina was used as the sequencer with high‐output mode and v4 chemistry. The sequencing run was single‐end with a read length of 101 bp and 1 mismatch allowed in demultiplexing.

The 3′ RNA sequences were processed using an integrated data analysis pipeline of the Lexogen QuantSeq 3′ mRNA‐Seq kit on the BlueBee Genomics platform (BlueBee Holding BV, Rijswijk, the Netherlands).

### 2.5 Identification of the Top 250 Most Variable Genes and Generation of Clustered Heatmap

Identification of the most variable genes and generation of a clustered heatmap was conducted as described previously [19]. Briefly, 250 genes with the greatest variations in expression levels among samples were identified by calculating gene‐wise variability from a logcounts matrix. The matrix, consisting of 250 genes and 28 samples, was subjected to hierarchical clustering in both dimensions using Pearson correlation as the distance measure and Ward. D2 as the linkage method. The resulting heatmap visually represents the expression levels of the 250 genes across the 28 samples. The genes and samples with distinct hotspots were selected based on the hotspot pattern in the heatmap. An enrichment analysis tool EnrichR (accessed 27 April 2024) [20, 21, 22] was utilized to investigate for enriched signalling pathways among each group of genes.

### 2.6 Virus Integration Analysis

Virus integration analysis was performed by using nf‐core/viralintegration pipeline [23] with the specific single‐end sequencing suitable code branch of version 0.1.1 [24]. Alignment was made against genome GRCh37 with viral genomes provided by the pipeline. Further parameter adjustments were done to adapt to QuantSeq read length and adapter trimming. The entire pipeline frozen at the version used in the study can be found at our GitHub page [25]. Downstream analysis of viral loads per sample was based on finding the top 5 viruses based on frac_covered value per sample as returned by the pipeline.

### 2.7 Histological Evaluation and Immunohistochemistry

Histopathological features were evaluated on haematoxylin–eosin–stained tumour sections by an experienced pathologist (Maria von Willebrand). Nuclear atypia (absent or mild, moderate, or severe), mitotic activity (high rate defined as > 10 mitosis/HPF), predominant cell morphology, presence of ductal or glandular differentiation, and presence of necrosis were systematically assessed, following criteria from a recent publication [26].

The presence of the *YAP1–NUTM1* fusion gene was assessed by IHC sensitive to YAP–NUT fusion protein detection [27]. Tumours were scored as NUT‐positive or ‐negative, and YAP1 staining intensity was categorized as absent (0), weak (1), or strong (2). The presence of the YAP1–NUT fusion protein was indicated when tumour samples showed concurrent positive nuclear staining for NUT and absent or weak staining for the C‐terminal region of YAP1, consistent with deletion of this region in the fusion protein [27].

The IHC staining patterns were further classified based on the classifications previously reported by Russell‐Goldman et al. [28]. In short, samples positive for NUT but negative or weak for YAP1 IHC are consistent with a *YAP1–NUTM1* fusion. Strong YAP1 staining with negative NUT staining reflects wild‐type expression. Negative or weak YAP1 combined with negative NUT suggests a *YAP1* fusion with a partner other than *NUTM1*. Finally, samples positive for NUT and strongly positive for YAP1 are consistent with a *NUTM1* fusion with a partner other than *YAP1*.

Additionally, IHC was used to detect HPV E7 oncoprotein expression for high‐risk HPV strains 16, 18, and 45 [29]. The technical details of IHCs are provided in Additional file 1.

### 2.8 Statistical Analysis

Associations between categorical and continuous variables were assessed using the Mann–Whitney *U* test. Relationships between categorical variables were analysed using Pearson's χ^2^ test or Fisher's exact test. All statistical analyses were conducted using SPSS Statistics software (IBM SPSS Statistics for Windows ver.29). A *p*‐value of less than 0.05 was considered indicative of statistical significance. P‐values in enrichment analysis were determined by a comparison between observed frequency of each term and the expected frequency [30]. *p*‐values < 0.05 were considered statistically significant.

## 3 Results

### 3.1 Patient Overview

An overview of patient and tumour characteristics is provided in Table 1. Mean age of EPC patients was significantly higher than that of EP patients (*p* = 0.033). The size of the excised tumour was larger in EPCs than in EPs (*p* = 0.023). There was no association between EPC or EP and sex (*p* = 1.000) or tumour location (*p* = 0.375). One of the EPCs had metastasized systemically at the time of surgery.

**TABLE 1:** Patient data of samples subjected to sequencing.

	EPC	EP	DPA
*N*	9	16	1
Age at first diagnosis (years)	75.9 (range 59–88)	61.7 (range 9–87)	22
Sex (M/F)	4/5	8/8	1/0
Tumour greatest diameter (mm)	56.7 (range 20–150)	21.3 (range 7–55)	5
no data (n)	3	3	
Metastasis at diagnosis (n)	1	0	0
no data (n)	1		
Survival (alive/deceased)	4/5	13/2	
no data (n)	0	1	1
Followup time (months)	141.5 (range 15–254)	172.5 (range 18–383)	
no data (n)	1	1	1
Location (n)			
head and neck	2	4	0
trunk	4	2	0
extremities	3	8	1
no data		2	0

*Note:* EPC: Eccrine porocarcinoma; EP: Eccrine poroma; DPA: Digital papillary adenocarcinoma.

### 3.2 Transcriptomic Overview of EPC and EP Based on Hierarchical Clustering

**FIGURE 1:**
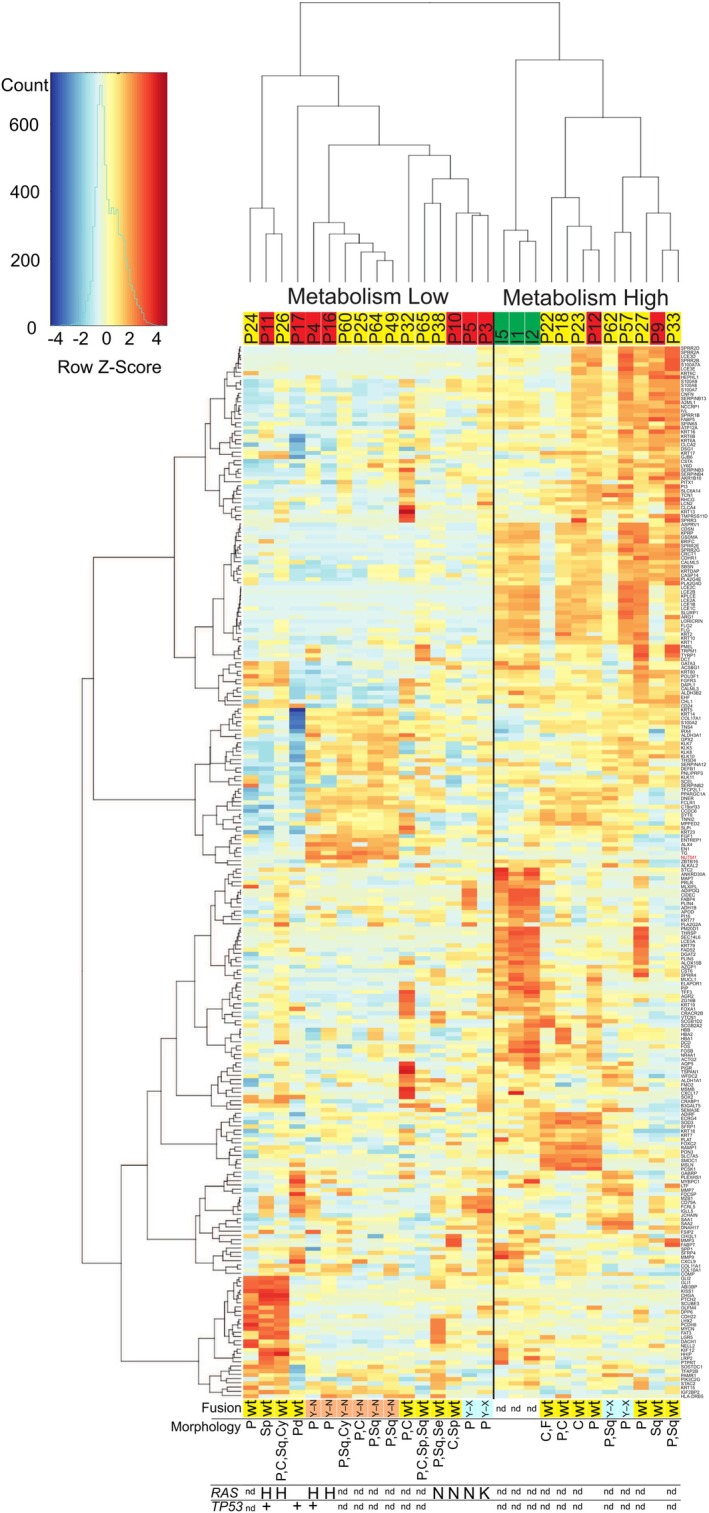
Heatmap displaying the top 250 most variable genes across healthy tissue, poroma, and porocarcinoma samples. The samples present in the heatmap were categorized into two groups (metabolism‐high and metabolism‐low) based on their gene expression patterns. Samples highlighted in green, yellow, and red represent healthy skin, EP, and EPC, respectively. Morphological characteristics, fusion status, and mutational profiles of *RAS* genes (*KRAS*, *NRAS*, and *HRAS*) and *TP53* are shown beneath the heatmap. Wt:Wild‐type; Y–N:YAP1–NUTM1 fusion; Y–X:YAP1–other gene than NUTM1 fusion; P: Poroid; Sp:Spindle; C:Clear cell; Sq:Squamoid; Cy:Cystic; Pd:Poorly differentiated; Se:Sebaceous; F:Follicular differentiation; H: HRAS mutation positive; N: NRAS mutation positive; K: KRAS mutation positive; nd: No data.

A clustered heatmap was created to display the top 250 most variable genes across the samples (Figure 1). The genes in the heatmap are listed from top to bottom in Additional file 2. No clear difference of transcriptomic profiles between EPCs and EPs was identified. The samples were stratified into two major clusters based on their gene‐expression profiles. The most notable difference between groups is the distinct expression of genes, which are significantly associated with gene‐pathway terms related to skin metabolism (including keratinization, melanogenesis, lipid metabolism) and the immune system (such as oncostatin M). Therefore, the two main clusters were named as a metabolism‐low and ‐high group based on their gene‐expression profiles. The metabolism‐low group included seven EPCs (77.8%) and nine EPs (56.3%). The metabolism‐high group contained normal skin samples in a separate branch, along with two EPCs (22.2%) and seven EPs (43.8%) in the second branch. The top 10 enriched pathways among the genes differentiating the metabolism‐low and ‐high groups are presented in Table 2 and all enriched pathways are presented in Additional file 3. There was no statistically significant association between metabolism‐high or ‐low groups and EPC or EP, sex, tumour location, age, or tumour size (all *p* > 0.05).

**TABLE 2:** Top 10 enriched pathways among genes differentiating the metabolism high and low groups.

Database	Term	*P*‐value	Adjusted P‐value
BioPlanet	Oncostatin M	2.2196441424174177E‐22	2.9299302679909916E‐20
	Hydrolysis of lysophosphatidylcholine (LPC)	5.031802927902886E‐4	0.028900662022061346
	Tyrosine metabolism	7.271534271965667E‐4	0.028900662022061346
	Melanogenesis	9.472776760529386E‐4	0.028900662022061346
	Amoebiasis	0.0010947220462902026	0.028900662022061346
	Acyl chain remodelling of phosphatidylinositol	0.002108808543204561	0.044942635193053825
	Acyl chain remodelling of phosphatidylserine	0.002383321563268006	0.044942635193053825
	Endogenous Toll‐like receptor signalling	0.004743270894726786	0.07331758007210236
	Corticotropin releasing hormone pathway	0.005141394197766799	0.07331758007210236
	Acyl chain remodelling of phosphatidylcholine	0.005554362126674421	0.07331758007210236
Reactome	Keratinization R‐HSA‐6805567	1.0581976448818804E‐43	1.5238046086299078E‐41
	Formation Of Cornified Envelope R‐HSA‐6809371	1.0391199123504648E‐37	7.481663368923347E‐36
	Developmental Biology R‐HSA‐1266738	1.6752537253311084E‐23	8.041217881589321E‐22
	Metal Sequestration By Antimicrobial Proteins R‐HSA‐6799990	7.810933303742631E‐12	2.8119359893473473E‐10
	Antimicrobial Peptides R‐HSA‐6803157	2.313409138459583E‐6	6.6626183187636E‐5
	Neutrophil Degranulation R‐HSA‐6798695	3.2306944262688276E‐5	7.753666623045186E‐4
	Melanin Biosynthesis R‐HSA‐5662702	1.8122211553393475E‐4	0.003727997805269515
	Hydrolysis Of LPC R‐HSA‐1483115	6.451389896168581E‐4	0.011612501813103446
	Innate Immune System R‐HSA‐168249	0.0015384157768353992	0.024614652429366387
	Acyl Chain Remodelling Of PI R‐HSA‐1482922	0.002383321563268006	0.02961709751164564
Wikipathway	Pancreatic Cancer Subtypes WP5390	2.7474280023472486E‐11	2.1155195618073816E‐9
	Vitamin D Receptor Pathway WP2877	1.5541789195027958E‐5	4.2797789518075977E‐4
	Burn Wound Healing WP5055	1.667446344860103E‐5	4.2797789518075977E‐4
	Hair Follicle Development Cytodifferentiation Part 3 Of 3 WP2840	3.6609687361769094E‐5	7.04736481714055E‐4
	GPR143 In Melanocytes And Retinal Pigment Epithelium Cells WP4941	2.865529734338402E‐4	0.004412915790881139
	Ras Signalling WP4223	0.0011905873840774212	0.015279204762326906
	Fatty Acid Transporters WP5061	0.0026737657475791204	0.029411423223370325
	Lipid Metabolism In Senescent Cells WP5149	0.0033018930995042783	0.031780721082728676
	Renin Angiotensin Aldosterone System RAAS WP4756	0.015379745356805696	0.13158226583044874
	IL 24 Signalling Pathway WP5413	0.01787464309354533	0.13763475182029905
KEGG	Oestrogen signalling pathway	2.7760092244822514E‐5	0.0018441255329713082
	IL‐17 signalling pathway	5.3119781397321337E‐5	0.0018441255329713082
	*Staphylococcus aureus* infection	5.5882591908221463E‐5	0.0018441255329713082
	Renin secretion	2.226201027372709E‐4	0.005509847542747455
	Tyrosine metabolism	4.946471577325671E‐4	0.009794013723104828
	GnRH signalling pathway	6.952776732748645E‐4	0.010810135751823838
	Inflammatory mediator regulation of TRP channels	8.463052347529611E‐4	0.010810135751823838
	Melanogenesis	9.472776760529386E‐4	0.010810135751823838
	Amoebiasis	9.827396138021671E‐4	0.010810135751823838
	Vascular smooth muscle contraction	0.002604939215151845	0.025788898230003264

*Note:* BioPlanet: BioPlanet 2019; KEGG: KEGG 2021 Human; WikiPathway: WikiPathway 2023 Human; Reactome: Reactome 2022.

The metabolism‐low group had one distinct hotspot in the expression heatmap, which included a subgroup consisting of one EPC (P11) and two EPs (P24 and P26). This subgroup showed elevated expression levels of genes associated with the Hedgehog signalling pathway, including *GLI1*, *GLI2*, *HHIP*, *LRP2*, and *PTCH2* (Additional file 4). Although the metabolism‐high group also included a group of four samples with elevated expression in genes such as *SOD3, FOXC2*, and *MSLN*, these genes did not share a common function or signalling pathway (Additional file 5). Compared with the tumour samples, the normal skin samples exhibited a distinctly higher expression of genes frequently associated with metabolic regulation (Additional file 6).

### 3.3 Detection of Fusion Proteins

The IHC results for YAP1‐NUTM1 fusion protein and the further classification of the staining patterns based on the classifications previously reported by Russell‐Goldman et al. [28]. are summarized in Additional file 7. In short, 22% of EPs and 22% of EPCs showed a staining pattern consistent with *YAP1*–*NUTM1* fusion. 22% of EPCs and 11% of EPs and exhibited a pattern suggestive of a fusion between *YAP1* and a partner other than *NUTM1*. No EPC or EP showed a pattern indicative of a *NUTM1* fusion partner other than *YAP1*.

The representative images of the YAP1 and NUT IHC are shown in Additional file 8. Morphological features, fusion status, and a subset of the mutational data provided in our previous study [18] for each sample are summarized in Figure 1 and the more detailed information is shown in Additional file 9. All samples with *YAP1–NUTM1* fusion pattern belonged to the metabolism‐low group. Notably, the expression of *NUTM1* was elevated in the heatmap across all samples with the *YAP1–NUTM1* fusion pattern. Many tumours consisted of more than one morphological pattern. All samples with *YAP1* fusion had poroid pattern and some of them also harboured squamoid pattern, while one sample contained clear cell pattern. All samples with no poroid pattern belonged to the group of samples with wild type staining pattern in YAP and NUT staining. All *RAS* and *TP53* mutations were found in the metabolism‐low group, although many EP samples lacked mutational data. There was no clear association between histological morphology, high mitotic activity, cytologic atypia, or necrosis and the metabolism‐high or low groups.

### 3.4 Virus Integration Analysis

**FIGURE 2:**
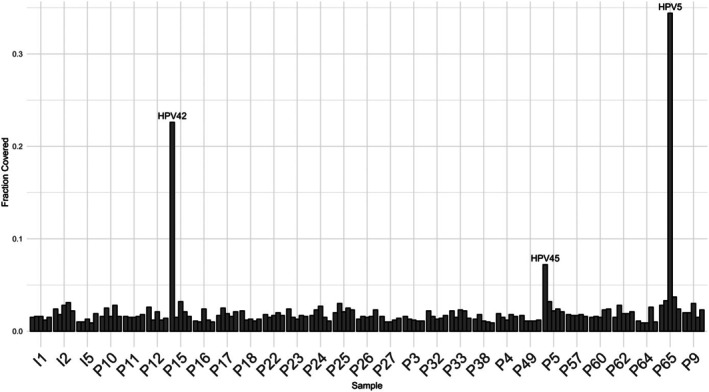
Virus load in poroma and porocarcinoma samples. Top 5 fraction covered values per sample with virus name shown only if frac covered value > 0.05, which were clearly higher than background levels.

To investigate the potential association between viruses and cancer development, we conducted a virus integration analysis using RNA sequencing data (Figure 2). A significantly high HPV42 load was observed in the DPA sample. As DPA is driven by HPV42 [14], this result demonstrates the validity of the analysis. One EP sample harboured a higher load of HPV5, and one EPC sample had a higher load of HPV45. Otherwise, no significant virus load was present in EPCs or EPs. HPV E7 oncoprotein expression was not detected in any samples (Additional file 10).

## 4 Discussion

This study aimed to characterize EPCs and EPs at the transcriptomic level, offering new insights into their transcriptomic similarities and differences. Our transcriptome analyses did not identify distinct profiles for EPCs and EPs. Instead, EPCs and EPs were classified into two distinct transcriptomic groups independent of tumour type. The most significant distinction between the groups was the differential expression of genes related to skin metabolism, including processes like keratinization, melanogenesis, and wound healing. This similarity supports the growing evidence on the indolent nature of many EPCs, demonstrating behavioural similarities with EPs [2]. Normal skin samples formed a distinct gene expression cluster (Figure 1), suggesting that EPCs and EPs have distinct transcriptomic features from normal skin. Interestingly, genes expressed at higher levels in normal skin were linked to oxidation pathways. Under normal conditions, cells generate energy through oxidative phosphorylation, whereas cancer cells predominantly rely on glycolysis [31]. The difference in the expression levels of oxidation‐related genes may therefore reflect the metabolic shift from normal skin to tumours. The metabolism‐high group included all normal skin samples and a subset of EPCs and EPs, indicating that these tumours are closer to normal skin in their transcriptome profile.

The expression level of genes associated with skin metabolism, such as keratinization, melanogenesis, and wound healing was significantly lower in the metabolism‐low group than in the metabolism‐high group. This suggests that there are likely gradual changes in these pathways as the skin transforms into EPs and EPCs. Although not statistically significant, the metabolism‐low group included more EPCs (77.8%) than the metabolism‐high group (22.2%). The genes with elevated expression in the metabolism‐high group included *KRT6A*, *KRT6B*, *KRT6C*, and *KRT16*, which encode proteins whose expression increases in response to tissue damage, cancerous cell transformation, and tumour growth [32]. The KRT16–KRT6 interaction enhances cell–cell and cell–matrix adhesion, thereby maintaining mechanical integrity [32]. Wound healing shares many mechanisms with cancer growth. For instance, activated angiogenesis is a common feature of both wound healing and cancer progression [33]. Additionally, re‐epithelialization during wound healing utilizes processes similar to those of tumour invasion, including basement membrane breakdown and interactions with non‐epithelial cells [34].

Genes associated with immune processes, such as oncostatin M, Toll‐like receptor signalling, antimicrobial peptides, innate immune system, and IL‐17 signalling pathway, were highly expressed in the metabolism‐high group. While viral infections are linked to some skin cancers, our virus integration analysis revealed that viruses are infrequently detected in EPCs and EPs. Although higher HPV45 and HPV5 loads were identified in two samples in our cohort, immunohistochemical staining for the HPV E7 oncoprotein was negative in all EPC and EP samples examined, arguing against involvement of high‐risk HPV 16, 18 or 45 strains in EPC or EP tumorigenesis, consistent with previous reports [15, 16, 17]. The immune system plays a key role in targeting cancer cells. For instance, tumour‐infiltrating lymphocytes are associated with a favourable prognosis in various cancers [35, 36]. Immunosuppression is a significant factor in the development of skin cancer [37]. Tumour cells can evade the immune system by several ways, such as downregulating major histocompatibility complex expression [38], shifting the balance of T helper cells from Th1 to Th2 [39], or expressing inhibitory molecules to immune cells, such as PD‐L1 [40]. In EPCs, both UV exposure and immunosuppression have been suggested as risk factors [41, 42].

Interestingly, the oncostatin M pathway was the most significantly enriched term in the BioPlanet database. Oncostatin M is a member of the interleukin‐6 family of cytokines, which activates various downstream transcription factors (such as STAT3 and STAT1) and MAPK signalling pathway proteins (such as extracellular‐regulated kinase 1/2, p38, and JNK) [43, 44]. Overexpression of oncostatin M is linked to aggressive behaviour in cancers, including squamous cell carcinoma of the skin [45], and hepatocellular carcinoma [46]. The Ras signalling pathway was also enriched in the metabolism‐high group, partially consistent with our previous findings showing that MAPK and Ras signalling pathways were enriched among genes mutated in EPCs [18]. Notably, recurrent mutations of HRAS have been reported in both EPCs and EPs [47, 48], suggesting that alterations in the Ras signalling pathway may drive the development of these tumours. Interestingly, metastatic EPCs are sensitive to the MEK inhibitor trametinib [7]. The metabolism‐high group also consisted of a subset of four samples with increased expression of the *MSLN* gene that encodes mesothelin, a cell‐surface protein emerging as a potential immunotherapeutic target in various cancers [49]. Various solid malignancies express mesothelin, such as ovarian carcinomas, pancreatic adenocarcinoma, endometrial carcinomas, malignant mesothelioma, and adenocarcinoma of the lung [50]. Further research is needed to explore and to develop therapies to target these proteins or signalling pathways in EP and EPC.

Interestingly, we also found elevated expression of Hedgehog (HH) pathway genes *GLI1*, *GLI2*, *HHIP*, *LRP2*, and *PTCH2* in two EPs and one EPC from the metabolism‐low group. The Hedgehog signalling pathway transmits signals from the cell membrane to the nucleus and plays an essential role in embryonic development and tissue homeostasis [51]. Key molecules in this pathway include Hedgehog ligands, such as sonic hedgehog, Indian hedgehog, and Desert hedgehog and the Patched (PTCH) receptors, Smoothened (SMO) receptor, and the glioma‐associated oncogene (GLI) transcription factors GLI1‐3. Normally, PTCH inhibits the activation of SMO, but when HH ligands bind to PTCH, this inhibition is disrupted, leading to activation of SMO and subsequent stimulation of GLI1 and GLI2, which promote transcription of target genes such as *CCNG1*, *MYC*, *BCL2*, *HHIP*, *SOX2*, and *PTCH1* [52]. Dysregulation of the Hedgehog signalling pathway has been implicated in the development of various skin cancers, including basal cell carcinoma [53] and Merkel cell carcinoma [54]. The SMO inhibitors vismodegib and sonidegib have already been approved by the FDA for treatment of basal cell carcinoma [55, 56]. Other inhibitors of the HH pathway, such as cyclopamine and GABT61, have shown potential in preclinical models of melanoma [57]. Further understanding of the involvement of the HH pathway in EPCs and EPs may lead to new treatment options targeting this pathway.

Sekine et al. identified *YAP1–MAML2*, *YAP1–NUTM1*, and *WWTR1–NUTM1* fusions in 68%, 20%, and 1% of EPs, and *YAP1–MAML2* and *YAP1–NUTM1* fusions in 9% and 55% of EPCs, respectively [8]. In our cohort, 22% of EPs and 22% of EPCs showed staining patterns consistent with *YAP1–NUTM1* fusion. Unlike Sekine et al., who found *YAP1–MAML2* ubiquitous in EPs, only 11% of our EPs displayed staining suggestive of a non‐*YAP1–NUTM1* fusion. NUT immunostaining performed well, as positivity closely matched elevated NUTM1 expression levels. However, these discrepancies suggest that correlation of YAP1 staining with sequence data is incomplete, likely due to the high specificity but lower sensitivity of YAP1 IHC. No EP or EPC showed evidence of *NUTM1* fusion with partners other than *YAP1*, consistent with previous findings [8, 28].

Prieto‐Granada et al. described *YAP1*‐fusion poroid tumours as having broad, bulbous projections of mixed basaloid and poroid cells, with squamatized cuticles, ductules, and uniform nuclei with grooves [58]. They also found few somatic mutations and a low mutation burden in tumours with *YAP1–NUTM1* or *YAP1–MAML2* fusions. In our series, the samples with *YAP1* fusions displayed poroid morphology, along with squamoid and clear cell morphology in some cases. We further compared the YAP1/NUT staining results with the mutation data obtained in our previous study [18]. Unfortunately, mutational data were unavailable for 11 of the 16 EPs, as only 5 had undergone exome sequencing in the previous study. In contrast, all EPCs had mutational data. Notably, *TP53* and *RAS* mutations were observed exclusively in the metabolism‐low group, suggesting that this group may represent a genomically more unstable or molecularly more advanced subtype, while tumours in the metabolism‐high group may develop through *TP53/RAS*‐independent mechanisms.

The two tumours reported by Prieto‐Granada et al. that lacked *YAP1* fusions were poroid hidradenoma and porocarcinoma in situ [58]. A more detailed description of their morphological features was not provided. In our study, several tumours with wild type YAP1 and NUT staining showed only non‐poroid morphology. Although no previous studies have reported findings identical to ours, our results may suggest morphological differences between tumours with and without *YAP1* fusions. The presence of hair follicle and sebaceous differentiation in EPCs and EPs with *PAK2* fusions supports an association between morphology and transcriptomic characteristics [11].

Our study has some limitations. The cohort size was limited due to the rarity of EPCs, which warrants a study of an independent cohort to verify our results. Because of the growing interest in fusion genes following the recent discovery of *YAP–MAML2*, *YAP1–NUTM1*, and *PAK2* fusion genes in EPCs and EPs [11], we attempted to identify fusion genes common to EPCs and EPs using RNA sequencing data and a standardized bioinformatics pipeline for RNA fusion detection. However, the analysis yielded multiple low‐confidence findings. This limitation was primarily due to the unavoidable degradation of RNA resulting from formalin fixation of the tumour specimen and sequencing library preparation based on the 3′ end of mRNA transcripts. Therefore, the association between transcriptomic profiles and fusion genes should be investigated in better‐preserved datasets.

In summary, this study offers important insights into the gene expression landscapes of EPCs and EPs, uncovering two major transcriptomic subtypes within these tumours while indicating that they differ from normal skin in their transcriptomic characteristics. The key genes that divided EPCs and EPs into the metabolism‐high and ‐low groups were associated with processes such as keratinization, melanogenesis, wound healing, and immune responses. Several signalling pathways, including Hedgehog and RAS–MAPK pathways, were dysregulated, which may offer opportunities for developing precision medicine approaches to treat EPC. The contribution of these pathways to EPC tumorigenesis and progression remains to be fully elucidated. All samples with the *YAP1–NUTM1* fusion pattern belonged to the metabolism‐low group and showed elevated *NUTM1* expression, supporting a correlation between heatmap‐based classification and fusion gene profiles. Advancing research through the development of EPC‐specific cell lines or in vivo models could enhance our understanding of this rare tumour and facilitate the discovery of targeted treatment strategies.

## Author Contributions

**Maya Puttonen:** conceptualization (equal), data curation (equal), formal analysis (equal), funding acquisition (equal), investigation (equal), methodology (equal), project administration (equal), visualization (equal), writing – original draft (equal), writing – review and editing (equal). **Sami Kilpinen:** formal analysis (equal), methodology (equal), visualization (equal), writing – review and editing (equal). **Kalle Ojala:** formal analysis (equal), methodology (equal), writing – review and editing (equal). **Tom Böhling:** conceptualization (equal), data curation (equal), funding acquisition (equal), methodology (equal), supervision (equal), writing – review and editing (equal). **Virve Koljonen:** conceptualization (equal), funding acquisition (equal), supervision (equal), writing – review and editing (equal). **Harri Sihto:** conceptualization (equal), funding acquisition (equal), investigation (equal), methodology (equal), project administration (equal), supervision (equal), writing – review and editing (equal). **Maria von Willebrand:** formal analysis (equal).

## Funding

This study was funded by the Jane and Aatos Erkko Foundation (4706174, T.B.), Medicinska Understödsföreningen Liv och Hälsa (4708936, T.B.), Finska läkaresällskapet (4709232, T.B.), Cancer Foundation Finland (4709194, H.S.), Helsinki University Central Hospital (HUCH) Competitive Research Fund (EVO) (TYH2020208, V.K.), and Emil Aaltonen Foundation (210179, M.P.).

## Ethics Statement

This study was approved by the institutional ethics committee of Helsinki University Central Hospital [HUS/358/2018].

## Conflicts of Interest

The authors declare no conflicts of interest.

## Supporting information


**Additional file 1** Word file (.docx). Technical details of immunohistochemistry.

**Additional file 2** Excel file (.xlsx). Heatmap genes and their Ensembl gene codes. The genes in the heatmap (Figure1) are listed from top to bottom.

**Additional file 3** Excel file (.xlsx). All enriched terms among the genes differentiating the metabolism‐low and ‐high groups.

**Additional file 4** Excel file (.xlsx). All enriched terms among the genes highly expressed in the expression hotspot in the metabolism‐low group vs. metabolism‐high group.

**Additional file 5** Excel file (.xlsx). All enriched terms among the genes highly expressed in the expression hotspot in the metabolism‐high group vs. metabolism‐low group.

**Additional file 6** Excel file (.xlsx). All enriched terms among the genes highly expressed in normal skin samples vs. tumour samples.

**Additional file 7** Excel file (.xlsx). The IHC results and the further classification of the staining patterns. Yellow = poroma (EP), red = porocarcinoma (EPC). The samples are shown in the same order as in the heatmap (Figure 1). The 2 EPCs (22%) and 4 EPs (22%) that stained positive for NUT was negative or weakly positive in YAP1 IHC, consistent with the YAP1–NUT fusion protein. All the samples with the staining pattern of *YAP1*–*NUTM1* belonged to the metabolism‐low group and more precisely to the same subgroup in the heatmap (Figure 1). 5 EPCs (56%) and 11 EPs (61%) exhibited a strong staining for YAP1 and a negative staining for NUT, consistent with wild‐type staining. 2 EPCs (22%) and 2 EPs (11%) were negative or weakly positive in YAP1 IHC and negative in NUT IHC, consistent with fusion between *YAP1* N‐terminus and other partner than *NUTM1*. No sample was positive for NUT and strongly positive for YAP1, consistent with fusion between *NUTM1* and a partner other than *YAP1*.

**Additional file 8** Scalable Vector Graphics file (.svg). The representative images of the YAP1 and NUT immunohistochemistry. Scale bar = 50 μm. For NUT expression, 0 = negative staining, 1 = positive staining. For YAP1 expression, 0 = negative staining, 1 = weak staining, 2 = strong staining. In the image showing negative YAP1 staining, YAP1 is expressed in the normal skin, whereas the tumour shows no detectable staining.

**Additional file 9** Excel file (.xlsx). Morphological features, fusion status, and a subset of the mutational data for each sample. Yellow = poroma (EP), red = porocarcinoma (EPC). Although most of the samples were of poroid morphology, part of the samples exhibited distinct morphologies such as spindle, clear cell, squamoid and poorly differentiated patterns. High mitotic activity was identified in 3 EPCs (33%), cytologic atypia was present in all EPCs and 3 EPs (19%), and areas of necrosis were identified in 2 EPCs (22%).

**Additional file 10** Scalable Vector Graphics file (.svg). The representative images of the HPV E7 immunohistochemistry. Scale bar = 50 μm. HPV E7 is expressed in the HPV18‐positive HeLa cells, whereas EPC shows no detectable staining.

## Data Availability

The datasets generated and/or analyzed during the current study are available from the Helsinki biobank (Project ID: HBP2018003) and the Finnish Clinical Biobank Tampere (pirha.fi/tampereen‐biopankki, Project ID: BB2018‐006) upon request. The codes used in this study are available on GitHub (https://github.com/Rare‐Cancers‐Research‐Group/Porocarcinoma).
