# Supplementary material for: RNA‐Sequencing Reveals Two Subgroups of Eccrine Porocarcinomas and Poromas
Source: J Cell Mol Med. 2026 Apr 14;30(8):e71140. doi: 10.1111/jcmm.71140 (PMC13079415; doi:10.1111/jcmm.71140)
Supplement: Supplementary file 1 — Additional file 1 Word file (.docx). Technical details of immunohistochemistry. [file JCMM-30-e71140-s007.docx]

**Additional file 1**

The following antibodies were used in IHC:
Rabbit monoclonal anti-YAP1 (clone D8H1X, 1:100 dilution, 40 min; Cell Signaling Technology, Danvers, MA)

Rabbit monoclonal anti-NUT (clone C52B1, 1:200 dilution, 1 hour; Cell Signaling Technology)

Mouse monoclonal anti-E7 antibody (clone 8E2, dilution 1:400, 1 hour; Abcam, Cambridge, UK).

Tissue microarrays were constructed using 1.0 mm core biopsies. Sections (4 µm) were deparaffinized in xylene, rehydrated through graded alcohols, and subjected to heat-induced antigen retrieval at 95°C for 15 minutes in EnVision FLEX Target Retrieval Solution, Low pH (Cat# K8005, DAKO, Glostrup, Denmark). Primary antibodies were diluted in Normal Antibody Diluent (Cat# BD09-500, WellMed, Duiven, The Netherlands). Detection of primary antibody binding was performed using the BrightVision 1-step HRP system (Cat# DPVM110HRP, WellMed) for 30 min and visualized with ImmPACT DAB (Cat# SK4105, Vector Laboratories, Burlingame, CA, USA) for 5 min, followed by counterstaining with Mayer’s hematoxylin (Cat# S3309, DAKO), dehydration through graded alcohol and xylene, and coverslipping.

An HPV18-positive HeLa cell line was processed into an FFPE cell block and used as a positive control for E7 IHC. Normal germ cells in the testis served as a positive control for NUT staining. YAP1 protein expression was evaluated across various tissues, with its absence in white blood cells confirming the specificity of the antibody used in the staining.
